# Development of a Mobile App (iCANSleep) to Treat Insomnia in Cancer Survivors: User-Centered Design Study

**DOI:** 10.2196/74387

**Published:** 2025-09-23

**Authors:** Sheila N Garland, Samlau Kutana, Katherine-Ann Piedalue, Rachel Lee, Joshua Rash, Gregory Cerallo

**Affiliations:** 1 Department of Psychology, Faculty of Science, Memorial University of Newfoundland, 232 Elizabeth Avenue, St. John's, NL, A1B3X9, Canada, 1 709-864-4897; 2 Discipline of Oncology, Faculty of Medicine, Memorial University of Newfoundland, St. John's, NL, Canada; 3 Beatrice Hunter Cancer Research Institute, Halifax, NS, Canada; 4 Sidekick Interactive, Montreal, QC, Canada

**Keywords:** insomnia, cancer survivors, cognitive behavioral therapy for insomnia, mHealth, mobile health, digital health, user-centered design, mobile app, needs assessment, usability testing

## Abstract

**Background:**

Insomnia affects the quality of life and health outcomes of cancer survivors. Cognitive behavioral therapy for insomnia (CBT-I) is an effective treatment for insomnia among cancer survivors, but it is not readily accessible due to the limited number of trained providers and the difficulties in providing care across wide geographical areas. Mobile health (mHealth) technologies represent a promising solution; however, these technologies are not tailored to the unique needs of cancer survivors.

**Objective:**

This study aimed to understand the needs and preferences of cancer survivors and test the usability of an evidence-based CBT-I smartphone app called iCANSleep that will be tailored and accessible to cancer survivors.

**Methods:**

A user-centered design (UCD) approach was applied, and cancer survivors were actively engaged in the app’s design, usability testing, and prototype refinement. In phase 1, semistructured interviews were conducted with a purposive sample of cancer survivors (n=20) to inform the design of the app and its content. In phase 2, iterative low- (n=8) and high-fidelity (n=7) usability testing was conducted with participants until no further recommendations for change were suggested.

**Results:**

Users suggested several defining characteristics, features, and desired functionalities, including a user-friendly and evidence-based design. They saw increased accessibility and simplicity as advantages of a mobile app but expressed some concerns about data security and losing the accountability that comes with in-person treatment. User testing highlighted the preference for images of real people and diverse stories over graphics and animated videos, and offered suggestions for enhanced navigation. The first iteration of the app was developed using the information gained during the needs assessment and usability testing. Feedback was integrated into the final prototype of the iCANSleep app, which will be tested for feasibility, acceptability, and efficacy.

**Conclusions:**

Cancer survivors desire an insomnia treatment app that is simple, user-friendly, evidence-based, convenient, and secure. The iCANSleep app represents the merging of mHealth principles and best practices with evidence-based insomnia care, allowing for an intervention with minimal access barriers related to cost, geography, and provider availability. Feasibility, acceptability, and efficacy of the intervention will be maximized by following a UCD framework involving the engagement of end users at every design stage.

## Introduction

Owing to increased screening, earlier detection, and advances in treatment, more people are living longer after cancer and with active disease [1,2]. The increasing number of cancer survivors (defined as individuals from the time of cancer diagnosis throughout the balance of life [3]) presents new challenges and opportunities for research to better address the long-lasting impacts of cancer and cancer treatment. Among the most common challenges of cancer survivorship is insomnia, which limits recovery [4] and quality of life [5]. Cancer survivors are approximately 3 times more likely to experience insomnia than the general population, and this insomnia can persist for up to 10 years after diagnosis [6-9]. Insomnia among cancer survivors is associated with many negative health consequences, such as increased pain [10], fatigue [11], perceived cognitive impairment [12,13], and reduced satisfaction with quality of life [5]. Moreover, disrupted sleep can have detrimental impacts on important objective health outcomes, including immune function, treatment response, and disease progression [4,14].

Cognitive behavioral therapy for insomnia (CBT-I) is recommended by the American Academy of Sleep Medicine and the American College of Physicians as the first-line treatment for insomnia [15,16] and has demonstrated effectiveness in several randomized controlled trials among cancer survivors [17-19] and the general population [20-22]. A meta-analysis of 15 trials reporting on 1461 cancer survivors indicated that participation in CBT-I resulted in clinically significant improvements in the time participants took to fall asleep, the amount of time spent awake while in bed, sleep quality, and self-reported insomnia severity [23]. CBT-I is a psychotherapy typically conducted over several weeks through regular sessions with a trained provider, which aims to address the perpetuating thoughts, emotions, and behaviors that contribute to insomnia [24]. Unfortunately, CBT-I remains inaccessible for most Canadians due in part to a shortage of appropriately trained clinicians [25]. Estimates suggest that there are only 752 CBT-I specialists worldwide, with only 5% residing in Canada. Access to qualified face-to-face providers in Canada is further complicated by the fact that a substantial proportion of residents live outside of metropolitan areas [26]. This means that cancer survivors in Canada, many of whom live in rural or remote areas, do not have adequate access to evidence-based treatment for insomnia. Additional barriers include a lack of insurance coverage, high treatment costs, and the difficulty of providing care across large rural areas. Owing to this lack of accessibility, pharmacological interventions, such as benzodiazepines and off-label hypnotics (eg, trazodone), have become the most frequently prescribed treatments for insomnia [27], despite well-documented risks that include tolerance and dependence, as well as withdrawal and rebound insomnia upon discontinuation.

Mobile health (mHealth), which is defined as the use of mobile and wireless technology (tablets, smartphones, etc) to support the improvement of health outcomes, health services, and health research, is a promising solution to the previously noted barriers to accessing CBT-I, especially given the high prevalence of smartphone ownership across age groups and socioeconomic status [28-30]. mHealth interventions are particularly well-suited to promote active engagement with and self-monitoring of chronic conditions, such as insomnia, and are considered by patients with cancer to be highly acceptable and feasible treatments [29]. Unfortunately, many of the currently available apps have been criticized for failing to adhere closely to evidence-based treatment recommendations [31]. iCANSleep will be the first evidence-based insomnia treatment smartphone app tailored to the unique needs of cancer survivors.

The objectives of this study were to (1) conduct a needs assessment with a sample of cancer survivors to inform the appearance, features, and content of the iCANSleep app, and (2) assess the low- and high-fidelity usability of the codeveloped iCANSleep app.

## Study Overview

### Design

The development of iCANSleep involved a user-centered design (UCD) process and followed a phased, sequential approach as recommended by the Medical Research Council Framework on the development of complex technology-based interventions [32]. UCD is an evidence-based framework for the development of technology-based medical interventions, which is informed by the needs and preferences of a specific group of end users [33]. A UCD process involves the engagement of end users at all stages of the development process, from needs assessment and app design to usability testing. Early involvement of cancer survivors in the design stages of the app allows the identification of features perceived as important to cancer survivors in a CBT-I app and is intended to address the common concerns with mHealth interventions (eg, early abandonment).

### Ethical Considerations

Ethical review and oversight were provided by the Health Research Ethics Authority of Newfoundland and Labrador. Approval was obtained for the needs assessment (HREB# 20222228) as well as both low- and high-fidelity testing (low fidelity: HREB# 20230515; high fidelity: HREB# 20231358). All interested participants were screened for eligibility and provided written informed consent. Once enrolled, participants were assigned a study number, and all subsequent information was tied to the study number to protect the identity of the participants. The researchers did not ask for names or any other identifying information during the interviews, but if participants provided such information, this information was not included in any transcripts. The recordings were tied to the study number and were securely erased after being transcribed and checked for accuracy. Questionnaires were emailed to the participants for completion via Qualtrics. Each participant who completed the interview or low/high-fidelity usability testing received a CAD $15 (US $11) digital gift card.

## Phase 1: User Needs Assessment

### Methods

#### Participants

Participants were purposively sampled, such that half of the sample reported having experience with in-person CBT-I and half reported no such experience. That was done to capture the perspectives of treatment-naïve and experienced cancer survivors. Cancer survivors who participated in past CBT-I trials were recruited directly from our mailing lists. Individuals without CBT-I experience responded to study advertisements shared through partnering networks and patient support organizations and were eligible to participate if they had (1) a previous cancer diagnosis of any type or stage, and (2) current reported insomnia as indicated by a score of >8 on the Insomnia Severity Index (ISI). A target sample size of 20 participants was selected based on the recognition that previous studies performing needs assessments through semistructured interviews have used similar sample sizes to achieve adequate thematic saturation, with further interviews providing limited novel information [34]. All participants who expressed interest in the study were interviewed. Reporting of the phase 1 assessment follows the Consolidated Criteria for Reporting Qualitative Research (COREQ) [35].

#### Procedure

An interview guide was developed by the research team based on a review of the literature and consultation with our industry stakeholders. End users provided feedback and improved the clarity of the interview guide before it was deployed. Eligible individuals participated in virtual interviews about their experiences with insomnia and cancer, their familiarity with smartphone technology, and their needs and preferences for an insomnia treatment smartphone app, using Webex. Interviews were conducted by SK (an experimental psychology master’s student with graduate training in qualitative research). Participants were introduced to the interviewer, explained the purpose of the interview, and offered an opportunity to ask questions prior to providing informed consent. Each interview lasted approximately 30 minutes and was attended by the interviewer and participant only. No repeat interviews were conducted. The semistructured interview guide is presented in Multimedia Appendix 1. Interviews were transcribed using NVivo software (Lumivero). Field notes were taken during the interviews, and transcripts were double-checked by the research team to ensure consistency and accuracy. Feasible recommendations for app design were drawn from the identified themes and translated into a set of functional requirements and design guidelines, which were used to develop the app prototype for usability testing. Transcripts were not returned to participants, and direct feedback on the findings was not provided. However, usability testing allowed for further user feedback.

#### Analysis

Interview transcripts were analyzed, and recurrent themes were identified through thematic analysis. Thematic analysis is a form of qualitative data analysis that is used to analyze patterns in the data and organize them into overarching themes [36]. The process involved 6 steps. In step 1, researchers read the interviews multiple times to familiarize themselves with the data. Then, in step 2, interviews were coded by 2 researchers, and the identified codes were discussed. This involved interpreting, identifying, and categorizing the major ideas and concepts in a passage (codes), and noticing which ideas and concepts are repeated or more central to the experience of interest. Codes are the smallest units of analysis that capture important features of the data, ranging in length from a single sentence or phrase to a paragraph. In steps 3 to 5, the codes were further categorized into overarching core ideas, known as themes, and iteratively grouped and refined by the research team. This process requires multiple reflexive iterations of transcript review, as categories are refined and restructured as more data are analyzed. The process is nonlinear as codes may be established and later updated or refined to fit with a new interpretation of the data. In step 6, the data were presented (*Results* section).

### Results

#### Participants

A total of 22 eligible participants responded to recruitment materials and were enrolled in the study. Technical issues with the audio recordings of 2 interviews rendered their transcripts unusable, and data from these 2 participants were excluded. The final sample of 20 participants (mean age 58.65, SD 15.68 years) included 14 women and 6 men. Of the 20 participants, 10 (50%) had experience receiving CBT-I in a virtual face-to-face format and 10 (50%) did not have previous experience or information about this intervention. Table 1 provides the demographic information of the sample, and Table 2 provides the cancer-specific information of the sample.

**Table 1.** Demographic and smartphone-related variables.

Variable	Value (N=20), n (%)	
Gender		
Female	14 (70)	
Male	6 (30)	
Age (years)		
18‐29	3 (15)	
30‐49	2 (10)	
50‐59	2 (10)	
60‐69	7 (35)	
≥70	6 (30)	
Province		
NL^a^	7 (35)	
NS^b^	6 (30)	
NB^c^	5 (20)	
BC^d^	2 (10)	
ON^e^	1 (5)	
Highest level of education		
Graduate degree or more	6 (30)	
Bachelor’s degree	10 (50)	
Some postsecondary education	4 (20)	
Employment status		
Retired	11 (55)	
Working full-time	7 (35)	
On disability support	2 (10)	
Relationship status		
Married or in a relationship	18 (90)	
Widowed	1 (5)	
Other	1 (5)	
Rural or urban status		
Rural	8 (40)	
Urban	12 (60)	
Insomnia treatment history		
Received CBT-I^f^ in the past	10 (50)	
Never received CBT-I	10 (50)	
Phone model		
iPhone	12 (60)	
Android	5 (25)	
Landline	3 (15)	

a NL: Newfoundland and Labrador.

b NS: Nova Scotia.

c NB: New Brunswick.

d BC: British Columbia.

e ON: Ontario.

f CBT-I: cognitive behavioral therapy for insomnia.

**Table 2.** Cancer and treatment-related variables.

Variable	Value (N=20), n (%)	
Cancer type		
Breast	7 (35)	
Melanoma	2 (10)	
Kidney	1 (5)	
Lung	1 (5)	
Blood	1 (5)	
Thyroid	1 (5)	
Ovarian	1 (5)	
Abdomen	1 (5)	
Lymph node	1 (5)	
Multiple	3 (15)	
Missing	1 (5)	
Cancer stage at diagnosis		
Stage 0	1 (5)	
Stage I	3 (15)	
Stage II	7 (35)	
Stage III	4 (20)	
Stage IV	2 (10)	
Current disease status		
No evidence of disease	16 (80)	
On treatment	2 (10)	
Living with metastatic disease	2 (10)	
Cancer treatment history^a^		
Surgery	17 (85)	
Radiation therapy	14 (70)	
Chemotherapy	11 (55)	
Hormone therapy	4 (20)	
Other	9 (45)	

a Proportion of the sample that received each of the treatments (each percentage is out of 100).

#### Cancer Survivors’ Recommendations for mHealth-Based Insomnia Treatment

Three major themes were identified from the participant interviews relating to what features and functionalities they would prefer in the app and the perceived advantages and disadvantages of an app-based insomnia treatment for cancer survivors. Each of these themes was further characterized by several subthemes to best capture key concepts.

##### Theme 1: Defining the Characteristics, Features, and Functionality of an Insomnia Treatment Smartphone App

###### Subtheme 1: User-Friendliness

Simplicity and ease of use were highly valued. Cancer survivors reported not wanting to spend too much time “figuring out” the app. Easy navigation included having a few large, clear buttons with apparent functions. CBT-I–naïve participants had mixed feelings about the user-friendliness of in-app reminders such as badges, sounds, and push notifications. While they did recognize the advantages of receiving timely reminders to complete certain tasks, they noted that overuse of these notifications would be perceived as annoying and would discourage long-term use of the app. A colorful, easy-to-understand data visualization was favored by these participants, particularly one that included information on the displayed variables and their relevance to sleep and insomnia.

Participants found that many apps they had used in the past imposed upon their daily life through irrelevant suggested content, excessive notifications, and intrusive advertising. Apps with heavy use of medical and technical jargon were considered difficult to understand and therefore less usable. This extended to apps that displayed data awkwardly or without explaining what the variables mean or how to understand the data display.

###### Subtheme 2: Evidence-Based Design

Cancer survivors reported being much more likely to use an app if it was endorsed by the medical community or their local cancer center. Participants liked that the content of the iCANSleep app would be designed by insomnia specialists and that it would deliver the recommended treatment for insomnia according to clinical practice guidelines. Cancer survivors who had no experience with CBT-I highly valued the ability to track their sleep within the app and receive individualized behavioral recommendations (eg, bed and rise time assignments) tailored to their own sleep data. This sentiment was echoed by participants who had experienced CBT-I in the past, and they also appreciated the ability to access curated evidence-based educational materials and resources within the app, rather than searching for similar information online, where it would be intertwined with information from unverified sources.

###### Subtheme 3: Desired Functionality

Participants with CBT-I experience felt that an app could consolidate several aspects of insomnia treatment. These participants saw the app as a convenient one-stop shop for receiving bed and rise time routine recommendations, tracking sleep diary indices as well as unique circumstances (events, stressors, and subjective sleep quality), seeking evidence-based information and resources on sleep, and receiving peer support through an anonymous forum. Those with CBT-I experience noted that sleep diaries would be more easily completed on a smartphone than using paper and pencils or a desktop computer. Participants also noted that the digital data display would be easier to understand at a glance than paper diaries and that this could be augmented through the thoughtful use of display colors, flags, and info bubbles.

Cancer survivors with and without CBT-I experience also expressed the desire to be able to connect their various wearable devices (eg, Fitbit and a smartwatch). Most participants agreed that the app’s ability to provide feedback and praise when certain tasks are completed or goals are achieved through badges, streaks, and other gamification features would augment their experience of the app and encourage its use. Participants with no CBT-I experience desired an insomnia treatment app that included guided meditation, calming sounds, and sleep stories to listen to while falling asleep in order to manage presleep stress and anxiety. This was not endorsed by participants with CBT-I experience.

### Theme 2: Advantages of Receiving Insomnia Treatment via a Mobile App

#### Subtheme 1: Accessibility

The primary advantage identified by cancer survivors for receiving insomnia treatment through a smartphone app was increased accessibility. Participants liked how an app for insomnia treatment could be accessed around the clock, without the need for scheduling appointments at a predetermined hour. Furthermore, the app could be accessed from any location, allowing treatment access when away from home, such as on vacation, and eliminating costs associated with travel to appointments, such as gas, parking, and childcare. One participant commented:

*I’ve always got my phone on me, so it doesn’t matter where I am if I’m camping or home or whatever. If I’m having trouble, I have techniques, I’ll have tools.*

Cancer survivors considered an insomnia treatment app that is available for free download to be highly advantageous compared to standard treatment channels.

*Something that’s actually accessible to people like a smartphone app makes it a lot easier for people to actually access care that they wouldn’t necessarily otherwise be able to access.*

One participant, speaking of the many ways an app would be more accessible for them, made the following statement:

*I think anything that gets it in the hands of people that doesn’t require a referral, or you know, $120 every time you go see them...more people are going to use it.*

#### Subtheme 2: Simplicity

Another major advantage mentioned by cancer survivors about receiving insomnia treatment through a smartphone app was simplicity. Participants with CBT-I experience felt that a properly designed smartphone app would simplify the daily process of tracking their sleep. One participant, speaking of the advantages of a digital sleep diary, made the following statement:

*It’s so much easier….You know, like especially where our phones are pretty much, for the most part, next to our beds, so you can quickly type it in….First thing I do, I check my email. I can easily plug in the numbers. Boom, it’s done.*

Participants also noted that the data display could facilitate the process of identifying trends in their own data. For some participants, using an app was also considered to be easier than finding an appropriate treatment provider. According to 1 participant, the ordeal of seeking out a new mental health professional was a big step to take. The participant commented as follows:

*…To finally go and see someone about it…it’s difficult when you go and you see a therapist three times and realize we’re not going to mesh. Like this isn’t going to work. And then the thought of having to go and repeat yourself all over again to another person and you know, that cycle. If there was an option of having an app that I could try, what’s the harm in trying?*

### Theme 3: Disadvantages of Receiving Insomnia Treatment via a Mobile App

#### Subtheme 1: Absence of a Therapeutic Relationship

Cancer survivors with experience receiving CBT-I from a provider, albeit virtually, were concerned that by receiving insomnia treatment through a smartphone app, they would miss out on the benefits of having a therapeutic relationship with a trained care professional. The ability to raise concerns or ask questions and receive answers from an experienced clinician was important to several participants, who highlighted the app’s relative lack of individual tailoring as a weakness. Some participants felt that the app’s ability to deliver cognitive therapy would be limited, and they were looking for a treatment that did more to “analyze” the causes and precipitants of their insomnia, rather than simply provide psychoeducation and behavioral recommendations. One participant commented as follows:

*I like face to face. It’s a personal preference. I think it allows me to ask questions that I can get answered as opposed to just putting in and they, the app, giving me back the information they think I want.*

#### Subtheme 2: Lack of Accountability

Participants with CBT-I experience reported that accountability to another human helped them remain engaged in past treatments, and they worried that engagement with an insomnia treatment app would be more difficult to maintain. These participants noted that the onus would be on the app user to engage with the app and maintain the daily sleep diary entries, and they felt that it would be easier to forget to complete the diaries or ignore any push notification reminders if there was no other human involved to keep them accountable.

*If I didn’t feel like it worked right away, I sometimes I have a tendency to give up early. It was helpful with a therapist because I felt that obligation to them. Whereas it might be easier to quit on an app because it’s an app and there’s nobody to disappoint.*

#### Subtheme 3: Reliance on Technology

Cancer survivors noted that treatment would only be available to people who own smartphones and who are located in a region with cell service. Some raised concerns that older participants or participants located in more rural areas would not be able to access the app. One participant commented as follows:

*There are people that because of where they live, don’t have the best reception with their phones and their apps. There are still a lot of places here in Newfoundland that are rural and don’t have the best reception.*

Participants also noted that certain events or circumstances might limit access to treatment, such as adverse weather conditions causing power or service outages. Some participants owned smartphones but expressed concerns that they were not familiar enough with the phone or with using apps to be confident in using an insomnia treatment app. One participant made the following statement:

*I’m not that old. I mean, my husband is in his early 80s. I’m going to be 75…I don’t feel I don’t feel old in any way, except when I pick up one of these [smartphones] and then or this [laptop computer] and then I feel old.*

#### Subtheme 4: Security, Privacy, and Data Ownership

Participants expressed distrust in the privacy and security of most apps, noting that many apps earn revenue through the sale of user data, and they mentioned that even those that do not explicitly sell user data are still vulnerable to unintentional data breaches. As a result, participants desired an insomnia treatment app that could be used while maintaining full anonymity and privacy. Participants with and without CBT-I experience highlighted the importance of being able to access their sleep data, both for privacy reasons and the ability to bring this easily understood information to another health care provider to augment the visit.

## Phase 2: Usability Testing of the iCANSleep App

### Methods

#### Basis of Usability Testing

The results from the needs assessment supported a consumer demand for a mobile insomnia intervention (eg, accessible and simple to use) tailored to cancer survivors and informed the defining characteristics (eg, user-friendly and evidence-based design), features (eg, gamification), and functionality (eg, built-in sleep diary and options for symptom tracking) of the app. The needs assessment also highlighted potential issues that would need to be addressed for the app to be acceptable to the users, including security, privacy, and data ownership. Potential users also identified possible limitations of an app-based intervention compared to traditional face-to-face treatment. This information can be used to identify those individuals who may or may not be best suited for an mHealth intervention.

Adhering to the principles of a UCD approach, usability testing of the iCANSleep app used an iterative process of testing the intervention’s user interface and prototype among a group of end users, collecting feedback, and using the feedback to redesign the prototype to meet users’ needs [37,38]. Current literature supports iterative usability testing to improve the likelihood of effectiveness in a technology-based intervention [33,39]. Both low- and high-fidelity usability testing cycles continued until saturation of recommendations was achieved. Low-fidelity usability testing uses simple design mock-ups of the app interface, usually presented as a digital wireframe, with feedback focusing primarily on basic functionality and design. This approach allows for the early identification of usability and design concerns before developing a prototype, making it a cost-effective way to implement participant feedback. Subsequently, high-fidelity testing uses a functioning prototype of the app, providing realistic user interactions. This final stage of usability testing helps to solidify the final design and refine the navigation of the app.

#### Participants

Individuals were eligible to participate if they had (1) a previous cancer diagnosis of any type or stage, and (2) current reported insomnia as indicated by a score of >8 on the ISI.

#### Procedure

##### Low-Fidelity Usability Testing

Low-fidelity usability testing involved showing participants a digital wireframe of the app’s user interface and soliciting feedback about aspects that are liked or disliked about the premise, design, and presentation of the app. Participants were further queried about suggestions for improvement. All interviews took place through Webex video conferencing software and were recorded. Researchers transcribed interview responses. Participants who participated in the low-fidelity usability testing received a CAD $15 (US $11) digital gift card.

##### High-Fidelity Usability Testing

High-fidelity usability testing took place following the development of a complete mock-up of the app. During a 30-minute in-person interview, participants were invited to explore the iCANSleep app and provide their impressions of its features and functions. Participants also completed a short questionnaire exploring their interest and willingness to use the app. Following a brief demonstration of the app, users were asked to navigate its contents and complete tasks (eg, make an entry in the sleep diary and locate a specific educational resource) while narrating aloud about the positive and negative aspects of app use. Audio of their voice and video of their hands as they navigated the app were recorded to help identify aspects of the app that were challenging to navigate. Additional information was generated from test participants through semistructured interviews assessing the app’s user interface design, content, and functions.

### Analysis

Data from the low- and high-fidelity usability testing interviews were transcribed and reviewed. Participants’ statements were examined for patterns that indicated usability issues (ie, tasks taking longer than expected). Similar feedback from multiple participants was interpreted to indicate systematic problems with the design, whereas outliers and less common feedback were taken to suggest issues that may affect specific user groups.

### Results

#### Low-Fidelity Usability Testing Results

Eight cancer survivors from across Canada viewed a digital wireframe of the app’s design and content and completed semistructured interviews. Participants (6 female and 2 male participants; mean age 53 years) were generally very impressed with the design and layout of the app. Most participants (6/8, 75%) were very interested in trying the app and reported that they would complete all or most of the app’s treatment program. Participants appreciated the integration of cancer-specific stories and material; the app’s simple, user-friendly design; the use of incentives and reinforcement; and the depth and breadth of its content. In the interviews, participants commented on the comprehensive sleep diary within the app and its apparent simplicity. Participants offered feedback on the presentation of the app and made recommendations to improve the usability of the interface. Suggestions were also made to streamline the onboarding and replace animations with real people. Based on the low-fidelity feedback, the research team removed the onboarding video and animations and replaced them with diverse patient stories to convey the necessary information.

#### High-Fidelity Usability Testing Results

Seven cancer survivors (4 female and 3 male participants) from St. John’s and the surrounding areas in Newfoundland attended a 30-minute in-person interview. Most participants (5/7, 71%) reported being very interested in trying the app, and 86% (6/7) indicated that they would complete the program. Additionally, 71% (5/7) reported that they would complete the program even if they did not see immediate results. All participants found the app easy to use and successfully completed all tasks and navigations. Suggestions for improvements included adding more “information” buttons throughout the app to provide additional context, increasing the size of the “information” buttons for better visibility, and organizing the bottom navigation menu. Participants also recommended improvements to the sleep diary, such as providing multiple options (eg, pain, washroom, pets, etc), along with an “other” button for the question “Did something influence your night?” instead of a text box response.

## Final Product

Based on the feedback from the user needs assessment and low- and high-fidelity user testing, we codeveloped an evidence-based insomnia treatment smartphone app for cancer survivors called iCANSleep (insomnia treatment for cancer survivors). Feedback from study participants in phases 1 and 2 was shared with the developers and user interface/user experience design members at our partner company, SideKick Interactive, to draw on their expertise and assess the appropriateness and feasibility of incorporating changes. The majority of the changes and updates to the app were driven by our participants to ensure that the needs of our target audience were prioritized. This self-guided insomnia treatment program is based on an established CBT-I treatment program [40] and incorporates evidence-based treatment components (described in Table 3).

**Table 3.** iCANSleep app module content overview.

Module	Summary of key components
Module 1: Getting to know your sleep	Introduction to CBT-I^a^; Creating sleep goals and the importance of self-monitoring using sleep diaries.
Module 2: Making your sleep more efficient	Sleep restriction; Creating a buffer zone; Users will receive their personalized sleep program based on sleep diary data from the past week.
Module 3: Making sleep your new habit	Stimulus control therapy; Reinforcing the connection between the bed and falling asleep by using the bed as a cue for sleep and restricting or avoiding other activities in bed.
Module 4: Relaxing into sleep	Relaxation techniques (progressive muscle relaxation, diaphragmatic breathing, etc); The role of relaxation techniques and how to schedule time for relaxation practice.
Module 5: Thinking straight about sleep	Cognitive restructuring; Challenging maladaptive beliefs about sleep, sleep problems, and sleep expectations, and creating realistic expectations and goals about sleep.
Module 6: Taking care of your sleep environment	Sleep hygiene psychoeducation.
Module 7: Sleeping with and beyond cancer	Users will receive strategies to help cope with relapses, strategies to instill motivation, and tools to maintain treatment gains over time.
Supplementary material	The extra resources provided to participants serve as supplementary material. The resources have been designed to address general and cancer-specific sleep questions or concerns raised by participants. Some examples include (1) Pain and sleep; (2) Sleep and exercise; (3) Hot flashes and sleep; (4) Should I take melatonin to help me sleep? and (5) How do cancer treatments affect sleep?

a CBT-I: cognitive behavioral therapy for insomnia.

**Figure 1.**
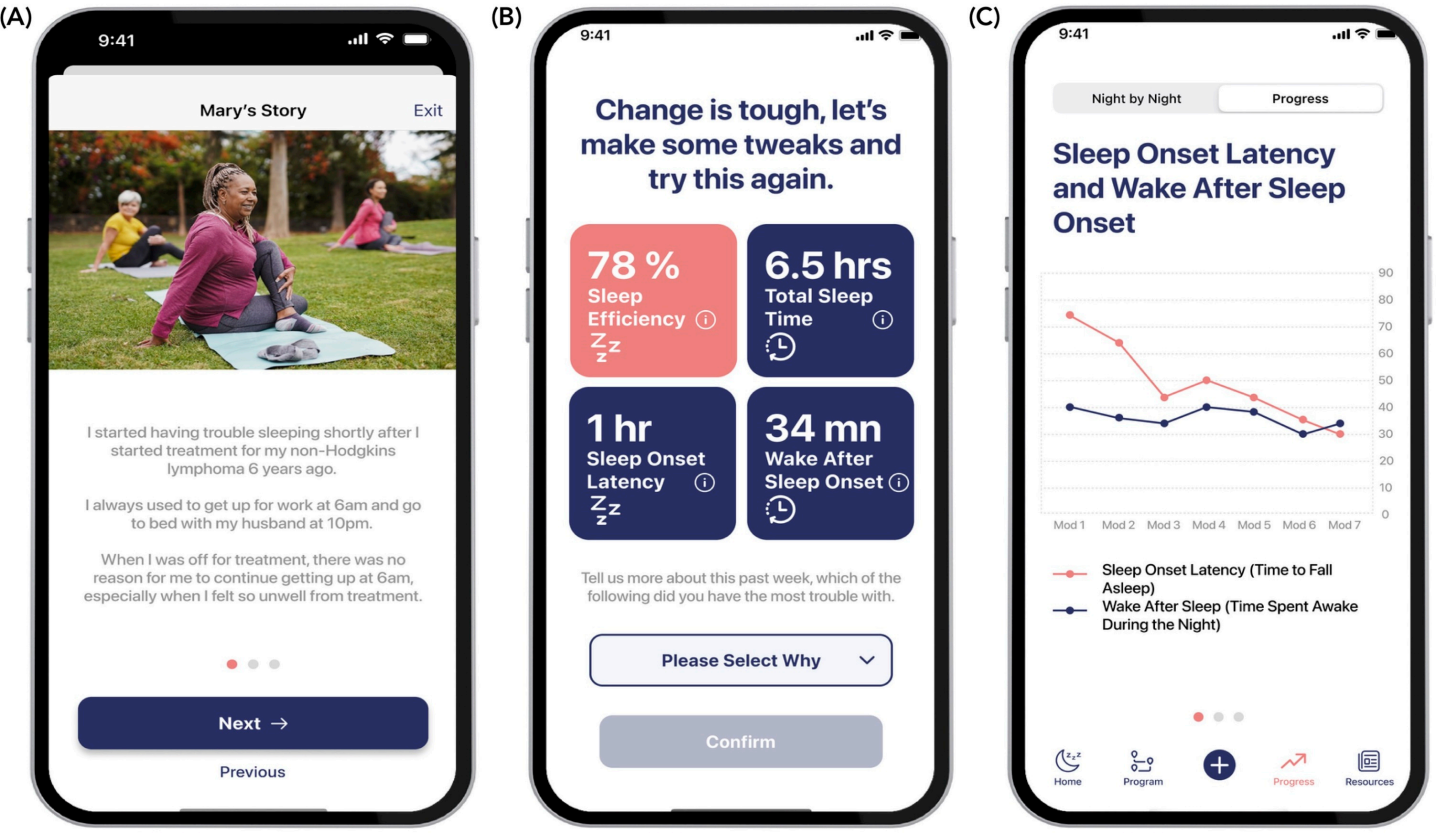
Screenshots of the user interface of the iCANSleep app showing (A) a story of a user with lived experience, (B) a weekly sleep summary, and (C) a weekly progress graph. Credit: Image created by the authors using content from Sabrina Bracher/Shutterstock.com.

The iCANSleep app allows for daily sleep tracking, provides tailored feedback for sleep improvement based on user-provided sleep data, and acts as an information resource for cancer survivors with insomnia. Figure 1 provides examples of the app’s appearance and features. As suggested by users, the app’s appearance is clean and uncluttered, with intuitive features and functions. The language is free of jargon, and any unfamiliar terms have an associated definition. Data from the sleep diary are presented to the user nightly each week, and weekly averages are available so that users can visualize their data and progress in the following areas: total sleep time, time spent in bed, sleep efficiency, sleep onset latency, amount of time spent awake at night, and number of awakenings.

Much attention was paid to the development of the sleep diary to ensure data accuracy. The development of the diary followed the recommendations of Shaffer et al [41], and it includes features to limit retrospective bias, promote user engagement, and provide data validation checks. Users must provide 5 days of sleep diary data in a 7-day period to allow for sufficient data to inform their individualized sleep recommendations and progress to the next module. Sleep efficiency cutoffs are used to inform the next week’s recommended sleep window. If the user’s sleep efficiency increases to 85% or higher, the app will offer praise, reinforce progress, and suggest that the user increase their sleep window by 30 minutes. If the user’s sleep efficiency is between 75% and 85%, the app will suggest that the user keep the recommended sleep window the same and address problem areas. If the user’s sleep efficiency is below 75%, the app will recommend that the user reduce their sleep window by 30 minutes, but never less than 5 hours. It will also provide support and suggest addressing challenges that the user might have encountered.

The lived experiences of cancer survivors are woven into the app onboarding so that users can “see themselves” in the app, and patient stories are used to demonstrate key treatment components. Notifications are limited to reminders to complete the sleep diary in the morning and a reminder to wind down before bed. Several gamification features are incorporated to encourage app engagement, including badges for sleep tracking streaks and awards for sleep improvements. As suggested, the app also features general and cancer-specific sleep information to address common challenges that users may face.

Users expressed concerns about the security, privacy, and ownership of their data. Based on this feedback, the research team is committed to collecting only absolutely necessary data (eg, sleep diary entries). The app also does not have any advertisement features for revenue generation and will not sell user information to third parties. To ensure the privacy of user data, a unique user identification number is generated upon user registration, and sleep data are stored in a secure SQL database that can only be accessed by approved members of the research team.

## Discussion

### Principal Findings

End users were involved in every phase of this research, including identifying priorities and preferences, development, and feasibility and usability testing, to ensure that the app is relevant and will address their unique concerns. The needs assessment revealed that cancer survivors wanted an app that is simple, user-friendly, evidence-based, convenient, secure, and a “one-stop shop” for all their insomnia-related needs. While all participants who tried the app prototype reported finding it easy to use and successfully navigating through the assigned tasks, they suggested valuable improvements for both organization and content. Participants with no CBT-I experience generally described user-friendly features of apps they had used in the past, requesting features like guided meditation and sleep sounds. Participants with CBT-I experience had a more concrete idea of what the treatment entailed, speaking more about how digital delivery would improve their experience, increase accessibility, and streamline treatment processes, such as completing the daily sleep diary. Phases 1 and 2 of our UCD approach resulted in the development of iCANSleep, an app that merges evidence-based insomnia treatment components with modern mHealth best practices for the delivery of insomnia care.

### Comparison With Prior Work

An evidence-based app for the treatment of insomnia is desperately needed to combat the multitude of apps that purport to help with sleep. Choi et al [42] reviewed nearly 2500 smartphone apps for sleep, concluding that the vast majority of sleep apps listed for download on common app stores fail to meet acceptable criteria for content, quality, or functionality in sleep self-management. To the untrained eye, finding an evidence-based sleep app through a simple search is thus a task akin to finding a needle in a haystack. Accordingly, there is a great opportunity for an evidence-based app focused on insomnia treatment. As recommended by the Medical Research Council Framework on the development of complex technology-based interventions [32], the next phase in the UCD and development of the iCANSleep app will involve testing the acceptability and feasibility of the app. This will be followed by a clinical trial to properly investigate the efficacy of the iCANSleep app for treating insomnia in Canadian cancer survivors.

### Limitations 

For receiving insomnia treatment through a smartphone app, cancer survivors highlighted some areas where they perceived disadvantages. These included a lack of a therapeutic relationship between the patient and clinician, a lack of accountability, and a reliance on technology. First, some participants felt that an insomnia treatment app would not have certain aspects of face-to-face treatments, such as individual tailoring and the ability to ask questions. This is a reasonable concern, and the exact trade-off between cost and effectiveness when it comes to therapist guidance versus automation of sleep therapies is currently being debated [43]. Despite past research showing increasing treatment effect sizes in therapies that include more intense therapist involvement [44], other research has shown that automating aspects of treatments can make them much more cost-efficient for producing improvements in sleep [45]. While iCANSleep will have a Frequently Asked Questions page as well as a repository of informational articles, it will not have the ability to flexibly answer individual inquiries or alter treatment based on specific personal circumstances. Moreover, the app might be able to provide some feedback and encouragement through gamification features (eg, badges and streaks), but an in-person clinician may be able to provide more personalized feedback. Second, participants also noted that there would be no clinician to hold them accountable, and the onus would be on themselves to engage with the app and follow its instructions. As such, iCANSleep will not entirely replace face-to-face individual treatment, and there remains a need for well-trained clinicians to offer CBT-I to support cancer survivorship. The iCANSleep app will expand treatment options and potentially allow for the prioritization of face-to-face interventions in patients for whom iCANSleep is not available or desirable. Third, participants recognized the potential for technological issues to arise, which would disrupt their ability to avail of the digital therapeutic service. Access is limited to patients who currently own a smartphone that can connect to cell phone towers, and a failure in any link of the chain (phone, power, internet service, and data) may cause the entire system to be inaccessible. Lastly, reliance on technology can leave patients open to digital risks, such as accidental breach of data, although these risks exist at varying levels for in-person treatments as well. During the development of iCANSleep, security and privacy were prioritized, resulting in a product that does not collect potentially identifiable information.

### Clinical Implications

Patients with cancer have identified that once cancer treatment is completed, their specialized care stops, despite still facing cancer-related mental and physical health challenges, including poor sleep. There are not enough CBT-I practitioners to address sleep concerns in cancer survivors. The development of iCANSleep aims to directly address these concerns by making follow-up survivorship care, specifically for sleep, accessible to all. After tests of efficacy are completed and future refinements are integrated, we plan to partner with cancer care authorities and support organizations to offer this treatment app to all cancer survivors.

## Supplementary material

10.2196/74387 Multimedia Appendix 1 Semistructured interview guide.

## Abbreviations:

CBT-I: cognitive behavioral therapy for insomnia
ISI: Insomnia Severity Index
mHealth: mobile health
UCD: user-centered design
