# Supplementary material for: Development of a Mobile App (iCANSleep) to Treat Insomnia in Cancer Survivors: User-Centered Design Study
Source: JMIR Cancer. 2025 Sep 23;11:e74387. doi: 10.2196/74387 (PMC12456873; doi:10.2196/74387)
Supplement: Multimedia Appendix 1 [file cancer-v11-e74387-s001.docx]

**Multimedia Appendix 1**

1. What do you see as the advantage(s) of receiving insomnia treatment through a mobile app?
2. What do you see as the disadvantage(s) of receiving insomnia treatment through a mobile app?
3. What are you currently doing to manage or improve your sleep that an app could help you with?
4. What would you like the tone of the app to be? Professional? Supportive? Entertaining?
5. If an app like iCANSleep had been available when you first started experiencing your sleep difficulty…
   1. Would you have used it?
      1. If yes, what is attractive to you about an app for insomnia treatment? Can you think of anything that would have discouraged you from using the app?
      2. If no, what would have stopped you from using the app? What could have made the app more attractive to you?
6. Often, cancer survivors with insomnia have other conditions such as anxiety, pain, or hot flashes that they feel interfere with sleep. What other conditions, ailments, or symptoms would you like iCANSleep to address?
7. How specific to the experiences of cancer survivors should the app be?
8. One advantage of a mobile app is the ability to include many different features; for example, iCANSleep could be designed to include things such as symptom tracking, peer support forums, information directories, “test your knowledge” quizzes, and more. What additional functions or features would you find helpful to include in iCANSleep?
